# Influence of tillage based crop establishment and residue management practices on soil quality indices and yield sustainability in rice-wheat cropping system of Eastern Indo-Gangetic Plains

**DOI:** 10.1016/j.still.2020.104841

**Published:** 2021-02

**Authors:** Kirti Saurabh, K.K. Rao, J.S. Mishra, Rakesh Kumar, S.P. Poonia, S.K Samal, H.S. Roy, A.K. Dubey, Anup Kumar Choubey, S. Mondal, B.P Bhatt, Mausam Verma, R.K. Malik

**Affiliations:** a ICAR- Research Complex for Eastern Region, Patna, 800 014, Bihar, India; b International Maize and Wheat Improvement Centre (CIMMYT), Regional Office, Patna, 800 014, Bihar, India; c ICAR- Mahatma Gandhi Integrated Farming Research Institute, Motihari, 845 429, Bihar, India; d ICAR- Indian Agricultural Statistics Research Institute, New Delhi, 110 012, India

**Keywords:** Rice-wheat cropping system, Zero tillage, Residue retention, Soil quality index

## Abstract

Rice-wheat cropping system (RWCS) is the most important system occupying around 26 M ha spread over the Indo Gangetic Plains in South Asia and China. Many long-term trials were led to assess the agronomic productivity and economic profitability of various combinations of conservation agricultural (CA) practices (zero tillage, residue management and crop establishment) in RWCS of Eastern Indo-Gangetic Plains (EIGP) of India. The purpose of this study was to investigate the best management practices involving different tillage-based crop establishment and residue retention techniques and their contribution to agricultural system sustainability through improvement in soil health by developing soil quality index (SQI). We have used SQI as an instrument based on physical [macro aggregate stability (MAS), available water capacity (AWC) and soil penetration resistance (SPR)], chemical [soil organic carbon (OC), available N, available P and available K] and biological [microbial biomass carbon (MBC), fluorescein diacetate (FDA) and dehydrogenase activity (DHA)] properties of soil, because these are very useful indicators of soil’s functions for agronomic productivity and soil fertility. Soil properties like MAS, OC, MBC, FDA and DHA were higher by 47, 18, 56, 48 and 53%, respectively, under ZTDSR-ZTW (T_7_: Zero-till direct seeded rice - Zero-till wheat) than RPTR-CTW (T_1_: Random puddled transplanted rice - Conventional till broadcasted wheat), at 0-10 cm. CA based treatment T_7_ also recorded lower SPR (126 N cm^-1^). SQI for different treatments were calculated by performing principal component analysis based on the total data set method. The higher system rice equivalent yield of 12.41 t ha^-1^ was observed at SQI value of 0.90 at 0-10 cm and 0.86 at 10-20 cm in T_7_. It can be concluded that crop residue retention on the surface with zero tillage is beneficial for the sustainability and productivity of the RWCS in EIGP of India.

## Highlights

• Soil physicochemical and biological properties were used for SQI development.
• Major parameters studied were FDA, DHA, MBC, SOC, Av. NPK, AWC, MAS and SPR.
• Highest SQI value of 0.90 was found in ZTDSR-ZTW at 0-10 cm soil depth.

## Graphical abstract

**Figure:**
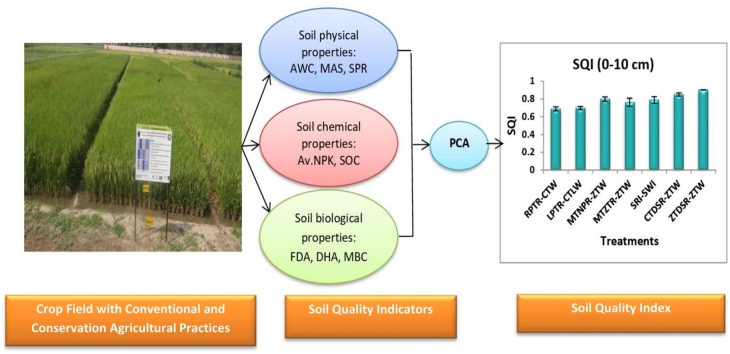

## 1 Introduction

Rice-wheat cropping system (RWCS) is the world's largest agricultural production system with approximately 12.3 M ha in India, 0.5 M ha in Nepal, 2.2 M ha in Pakistan and 0.8 M ha in Bangladesh and approximately 85 percent of this area falls into Indo-Gangetic Plains (IGPs) (Mahajan et al., 2009). IGPs of India is considered as the major food bowl of the country. Most of the IGP area is occupied by the rice-wheat rotation which constitutes about 40% of the agricultural area followed by other cropping systems like rice-fallow-fallow and maize-wheat (Panigrahy et al., 2010). Though this cropping system is occupying a large area of IGP, the sustainability of this system has emerged as a burning issue among the researchers due to stagnation of yields of both rice and wheat. Indiscriminate ploughing, residue removal/burning, unbalanced use of chemical fertilizers and extractive farming exacerbated the deterioration of soil and atmosphere quality (Montgomery, 2007), soil organic carbon (SOC) pool, soil biodiversity, compaction, increased runoff, accelerated erosion loss of nutrients, carbon and water from the ecosystem, loss of soil resilience and decrease in ecosystem services (Lal, 2015). Therefore, adoption of the balanced cropping practices, such as conservation tillage (Zhang et al., 2015), recycling of crop residues (CRs) (Devi et al., 2017), application of manure (Tadesse et al., 2013) and farmland fallow (Liu et al., 2012), would be much sought after in the present circumstances for improving the soil quality, ecosystem function and yield sustainability of RWCS. Conversion of conventional agricultural practices to conservation has great potential for increasing the soil quality through the presence of C substrate, increasing soil biodiversity, MBC, soil aggregation and improving water and nutrient use efficiency (Tisdall and Oades, 1982; Ghosh et al., 2015; Li et al., 2018).

Many of the issues of agricultural sustainability are associated with soil quality. Thus, its assessment and the way it changes with time is a significant pointer of whether the farming system is sustainable (Masto et al., 2007). Soil quality is an integration of physical, chemical and biological properties of the soil, which can easily change in response vis-à-vis changes in soil conditions (Brejda et al., 2000). Different types of land use and agricultural management practices are mainly responsible for the change in soil fertility and productivity, which lead to changes in the properties of the soil (Islam and Weil, 2000; Sanchez-Maranon et al., 2002). Integration of several soil properties to soil quality indices can provide a better picture of soil quality than individual parameters. Further one should select soil indicators that interact synergistically which can help in measuring the soil quality and stability under different crop cultivation practices. Doran, 2002 defined soil quality as the capacity of a soil to function and promote plant and animal productivity, and maintain or enhance water and air quality. Maintenance and promotion of soil quality is, therefore, a fundamental requirement to ensure ecosystem sustainability. As soil quality is often associated with management practices as well to soil characteristics. In the early 1990s, therefore, a mathematical or statistical framework was proposed to estimate the SQI (Doran et al., 1994; Karlen et al., 1997). The SQI was evaluated in order to focus the management objectives not only on productivity per se, which can lead to soil degradation (Larson and Pierce, 1991), but also on environmental issues. A suitable SQI can, therefore, have three component objectives: environmental quality, agricultural sustainability and socio-economic viability (Andrews et al., 2002). SQI helps to evaluate the quality of the soil of a given site or ecosystem and allows comparisons between conditions at field level for different land use and management practices.

To date, plentiful studies have focused on the impacts of different crop establishment, tillage and residue management on soil properties and crop productivity (Mbuthia et al., 2015; Shekhawat et al., 2016; Nandan et al., 2018; Singh et al., 2018). Literatures are also available on various soil parameters, particularly physico-chemical and few on biological but in isolation under CA based management system in IGP. However, the information about identifying the best conservation agriculture system to achieve the optimal yield based on the development of SQI having all three soil properties i.e. physico-chemical and biological in EIGP is limited. Therefore, the present study was conducted with the objectives to assess the impacts of different crop management practices on soil quality and fertility. The hypothesis tested was that the zero till system with one third CR retained on the soil surface and inclusion of legume in RWCS will improve soil physical, chemical and biological properties owing to high SQI and system productivity.

## 2 Materials and methods

### 2.1 Experimental site

Soil samples were collected from the ongoing experiment of institute farm, Patna (25 °24.912’ N and 85 °03.536’ E) during 2015-2018, situated in the subtropical humid climate of Eastern IGP, with an average annual rainfall of 1130 mm, mean minimum temperatures of 7-9 °C in January, mean maximum temperatures of 36-41 °C in May, and relative humidity of 60–90% throughout the year. The initial physicochemical properties of the experimental field soil having silty loam texture were as follows: pH- 7.2; Walkley-Black C- 0.6%; available N- 172 kg ha^-1^; available P- 12.9 kg ha^-1^, available K- 137 kg ha^-1^; BD- 1.67 Mg m^-3^ and DTPA extractable Fe, Zn and Cu were 14.4, 0.93 and 3.63 mg kg^-1^, respectively.

The experiment was carried out in a randomized block design with three replications with individual plots measured 20 m x 8 m. Treatments (T_1_-T_7_) were based on tillage (wet tillage or puddling, dry tillage and zero-tillage), crop residue management (either incorporated or retained on the soil surface) and establishment methods (transplanting, direct-seeding or drill seeding) in rice and wheat cropping system. Description of experimental treatments with cropping details is given in Table 1.

**Table 1:** Crop establishment, tillage and residue management practices followed in rice-wheat-greengram cropping system.

S.No.	Treatment	Crop establishment methods	Tillage	Crop residue management
1	**T_1_**: Random puddled transplanted rice (RPTR) - Conventional till broadcasted wheat (CTW)-ZT greengram	Rice: random transplanting Wheat: broadcasting Greengram: drill seeding	Rice: puddling (2 dry harrowing + 1 wet tillage + rotavator) Wheat: CT (2 harrowing + rotavator) Greengram: ZT	Rice: one-third incorporated Wheat: one-third retained on soil surface Greengram: full incorporated
2	**T_2_**: Puddled line transplanted rice (LPTR)- Conventional tillage line sown wheat (CTLW) -ZT greengram	Rice: line transplanting (0.2 m X 0.15 m) Wheat: line sowing Greengram: drill seeding	Rice: puddling (2 dry harrowing + 1 wet tillage + rotavator) Wheat: CT (2 harrowing + rotavator) Greengram: ZT	Rice: one-third incorporated Wheat: one-third retained on soil surface Greengram: full incorporated
3	**T_3_**: Machine transplanted non-puddled rice (MTNPR) - Zero-till wheat (ZTW) -ZT greengram	Rice: machine transplanting (0.2 m × 0.25 m) Wheat: drill seeding Greengram: drill seeding	Rice: unpuddled (2 harrowing + rotavator) Wheat: ZT Greengram: ZT	Rice: one-third retained on soil surface Wheat: one-third retained on soil surface Greengram: full incorporated
4	**T_4_**: Machine transplanted zero-till rice (MTZTR) - ZTW-ZT greengram	Rice: machine transplanting Wheat: drill seeding Greengram: drill seeding	Rice: ZT Wheat: ZT Greengram: ZT	Rice: one-third retained on soil surface Wheat: one-third retained on soil surface Greengram: removed
5	**T_5_**: System of rice intensification (SRI) - System of wheat intensification (SWI) -ZT greengram	Rice: transplanting (0.25 m × 0.25 m) Wheat: sowing (0.225 m × 0.225 m) Greengram: drill seeding	Rice: puddling (2 dry harrowing + 1 wet tillage + rotavator) Wheat: CT (2 harrowing + rotavator) Greengram: ZT	Rice: one-third incorporated Wheat: one-third retained on soil surface Greengram: full incorporated
6	**T_6_**: Conventional till direct seeded rice (CTDSR) - ZTW-ZT greengram	Rice: drill seeding Wheat: drill seeding Greengram: drill seeding	Rice: CT (2 harrowing + rotavator) Wheat: ZT Greengram: ZT	Rice: one-third retained on soil surface Wheat: one-third retained on soil surface Greengram: full incorporated
7	**T_7_**: Zero-till direct seeded rice (ZTDSR) - ZTW-ZT Greengram	Rice: drill seeding Wheat: drill seeding Greengram: drill seeding	Rice: ZT Wheat: ZT Greengram: ZT	Rice: one-third retained on soil surface Wheat: one-third retained on soil surface Greengram: surface mulch killed by glyphosate application

Rice cultivar “Arize 6444” was sown in the nursery at seed rate 20 kg ha^-1^ and 5 kg ha^-1^ for transplanting (T_1_, T_2_, T_3_ and T_4_) and SRI (T_5_), respectively, while for direct-seeding it was drill seeded at 30 kg ha^-1^ (T_6_ and T_7_). Direct seeded rice (DSR) was sown in the first week of June using Happy seeder and transplanting was done in the last week of June. Twenty-five days old 2-3 seedlings per hill were transplanted in T_1_, T_2_, T_3_ and T_4_ whereas; twelve days old single seedling per hill was transplanted in SRI treatment. After rice harvest, wheat cultivar “HD 2967” was sown at the rate of 100 kg ha^-1^ for ZT and CT i.e. (T_1_, T_2_, T_3_ and T_4_, T_6_ and T_7_) and at the rate of 25 kg ha^-1^ in SWI (T_5_) on the first week of November. Just after the wheat harvesting, greengram (Pusa Vishal) was sown at the rate of 25 kg ha^-1^. In rice and wheat, fertilizers were applied at recommended dose: 120 kg N ha^-1^, 60 kg P_2_O_5_ ha^-1^ and 40 kg K_2_O ha^-1^. For greengram fertilizers, 20 kg N ha^-1^ and 50 kg P_2_O_5_ ha^-1^ were applied. At the time of seeding in DSR, a full dose of P and K and 1/3-dose of N were applied using a Happy seeder and were broadcasted manually at the time of transplantation in rice. The remaining N was applied in two equal splits at maximum tillering and panicle/ear emergence.

In ZT plots, pre-plant use of glyphosate at 0.9 kg ha^-1^ killed existing weeds before seeding rice and wheat. During the growing season, the plots were kept free of weeds using herbicides. In transplanted rice, butachlor at 1.5 kg ha^−1^ was applied as pre-emergence within 2 days of transplanting, whereas, in direct-seeded rice, bispyribac sodium at 80 g ha^−1^ was applied at 25 days after sowing (DAS). Moreover, hand-weeding was also necessary to keep the plots weed-free. Weeds were controlled in wheat using Total (sulfosuluron 75% WG + met sulfosulfuron methyl 5% WG) @ 40 g ha^-1^ after 25-30 DAS. Pendimethalin at the rate of 1 kg ha^-1^ was applied in greengram as pre-emergence (2-3 DAS).

### 2.2 Soil sampling and lab analysis

After the completion of the 4^th^ year of the experiment, soil samples were collected from 0-10 and 10-20 cm soil depth. A composite sample was prepared by collecting soils from five randomly selected points of plots with soil augur. A fresh subsample for each treatment was stored at 4 °C for subsequent analysis of biological properties and rest soils were air-dried in the lab and subjected to pass through 2 mm sieve for analyzing chemical and physical properties of soil. The organic carbon (OC) content was determined by dichromate oxidation of the sample and subsequent titration with ferrous ammonium sulfate (Walkley and Black, 1934). The available N content was obtained by the Kjeldahl method followed by titration with diluted sulfuric acid (Subbiah and Asija, 1956). Available P content was determined by the NaHCO_3_ -ascorbic acid method (Watanabe and Olsen, 1965), and available K content with the ammonium acetate method using a ﬂame photometer (Hanway and Heidel, 1952).

Soil penetration resistance was measured using a hand penetrometer (Mondal et al., 2016). The pressure plate method was used for determining the water contents held at -0.33 bar and-15 bar matric potential (Piper, 1966). The plant available water content (AWC) was calculated as the diﬀ ;erence between gravimetric water content at ﬁeld capacity (-0.33 bar) and permanent wilting point (-15 bar). Aggregate stability was performed using a wet sieving technique (AL-Maliki and Scullion, 2013).

Microbial biomass carbon (MBC) in soil was measured by the method of Nunan et al. (1998) with some modiﬁcation as mentioned in Parihara et al. (2016). Dehydrogenase activity in soil was estimated by the procedure outlined by Tabatabai and Bremner (1969). Fluorescein diacetate (FDA) hydrolytic activity in soil was determined using the procedure mentioned by Green et al. (2006).

### 2.3 System productivity or system rice equivalent yield (SREY)

SREY, the total productivity of rice, wheat and greengram as a whole, was reported for the 4^th^ year of the cropping system. The calculation of SREY was done as described by Bohra and Kumar (2015) and the yields of all non-rice crops were converted to rice equivalent yield using the equation given below for the estimation of SREY.

$$Rice\;equivalent\;of\;non\;rice\;crop\;yield=\frac{Non\;rice\;crop\;yield\;(t/ha)\;\times \;MSP\;of\;non-rice\;crop\;(INR/kg)}{MSP\;of\;rice\;crop\;(INR/kg)}$$

### 2.4 Soil quality indexing

Three steps were followed in the development of SQI: 1) the total data set (TDS) method was selected and all 10 variables were taken into account; 2) the indicators were scored, and 3) the scores were integrated into final SQI. Principal component analysis (PCA) was carried out using all observations of the measured soil properties and the weight of each TDS indicator was calculated by communalities, which was equal to the ratio of its communality divided by the sum of other communalities of all TDS indicators (Liu et al., 2017). To get a clear cut knowledge on the variations in soil quality indicators in a similar type of soils under various distinct management systems is necessary to convert the raw data of soil quality indicators into unitless numerical scores. So, the actual values of soil properties were normalized to obtain a score of an individual parameter (X_i_) by the relation X_i_ = X_o_/X_max_ for ‘more is better’ properties and X_i_ = X_min_/X_o_ for ‘less is better’ properties (Das et al., 2016). In this study, the soil penetration resistance was considered as ‘less is better’ and all other parameters were treated as ‘more is better’. Subsequently, the weighted variable scores for each observation were integrated for two soil depths 0-10 and 10-20 cm by using the following equation:

(1) $$\text{S}\text{Q}\text{I}=\;\sum_{i=1}^{n}Wi\times Si$$

Where Wi is the weight of each indicator derived from PCA and Si is the score for the variable.

### 2.5 Statistical analysis

Analysis of variance (ANOVA) was performed to determine the effects of different treatments on soil quality parameters. Means were separated using Duncan’s multiple range test (DMRT) with a 0.05 significance level using SPSS software. Contrast analysis of different treatment means groups were done using SAS. Principal Components Analysis (PCA) (XLStat 7.5, Addinsoft) was used to determine SQI.

## 3 Results

### 3.1 Soil physical indicators

Coarser aggregates serve as a worthy indicator of changes in soil quality, in particular, soil porosity, which aﬀ ;ects aeration and water retention and are more vulnerable to external processes, such as tillage. In this study, MAS was found to be significantly (*p* < 0.05) affected by the tillage and residue management based treatments (Table 3). The T_7_ treatment showed higher MAS than any other treatments at each depth (Table 2). The MAS in different treatments followed the order of T_7_ > T_6_ > T_3_ > T_4_ > T_5_ > T_1_ > T_2_ at 0-20 cm soil surface. The contrast analysis depicted a significant difference between CT and RT, CT and ZT but no significant difference was observed between RT and ZT treatment means at 10-20 cm soil depth (Table 3). After 4^th^ year of study, aggregate stability varied between 40.1 to 59.2% at 0-10 cm and 49.8 to 69.9 % at 10-20 cm soil profile depth. Furthermore, the increase in MAS in 0-10 cm was 47% under T_7_ compared to T_1_.

**Table 3:** Analysis of variance for various soil properties influenced by different residue and tillage based crop establishment treatments at 0-10 cm and 10-20 cm soil layer.

contrast	FDA (mg fluorescein kg^-1^ soil hr^-1^)	DHA (μg TPF g ^−1^ soil h^−1^)	MBC (μg C g^-1^ soils)	Av. N (kg ha^-1^)	Av. P (kg ha^-1^)	Av. K (kg ha^-1^)	O.C (%)	MAS (%)	AWC (%)	SPR (N cm^-1^)
**0-10 cm**	P > F									
**R_i_ vs R_r_**	**	**	**	ns	ns	ns	**	**	**	**
**CT vs RT**	**	**	**	ns	ns	ns	**	**	**	**
**CT vs ZT**	**	**	**	ns	ns	ns	**	**	**	**
**RT vs ZT**	**	**	**	ns	ns	ns	**	**	**	**
**10-20 cm**										
**R_i_ vs R_r_**	**	**	**	**	ns	ns	**	**	**	**
**CT vs RT**	**	**	**	**	ns	ns	**	**	**	**
**CT vs ZT**	**	**	**	**	**	ns	ns	**	**	**
**RT vs ZT**	ns	ns	ns	**	**	**	ns	ns	ns	**

** *p* < 0.05

Contrasts analysis were used to test differences among treatment means grouped into residue incorporated (Ri: mean of RPTR-CTW, LPTR-CTLW and SRI-SWI), residue retained (Rr: mean of MTNPR-ZTW, MTZTR-ZTW, CTDSR-ZTW and ZTDSR-ZTW)) based on rice residue management and tillage practice during rice and wheat establishment i.e. conventional tillage (CT: mean of RPTR-CTW, LPTR-CTLW and SRI-SWI), reduced tillage (RT: mean of MTNPR-ZTW and CTDSR-ZTW) and zero tillage (ZT: mean of : MTZTR-ZTW and ZTDSR-ZTW)

**Table 2:** Soil properties as influenced by diﬀ ;erent treatments in the 0–10 and 10–20 cm soil layers of the rice-wheat-greengram cropping system.

Soil layer	Treatment	FDA (mg fluorescein kg^-1^ soil hr^-1^)	DHA (μg TPF g ^−1^ soil h^−1^)	MBC (μg C g^-1^ soils)	Av. N (kg ha^-1^)	Av. P (kg ha^-1^)	Av. K (kg ha^-1^)	O.C (%)	MAS (%)	AWC (%)	SPR (N cm^-1^)
0-10 cm	T_1_: RPTR-CTW	37.4d	12.0c	67.8c	154.5b	11.2bc	143.5c	0.65c	40.1c	15.5c	177.7a
	T_2_: LPTR-CTLW	39.5d	12.5c	71.8c	158.7b	13.4ab	148.3c	0.66c	40.8c	14.9c	175.8a
	T_3_: MTNPR-ZTW	47.2b	16.0ab	98.1ab	175.4ab	13.3ab	181.1a	0.74ab	57.2ab	19.0ab	159.4ab
	T_4_: MTZTR-ZTW	43.4bc	15.8ab	94.0b	157.4b	11.8bc	160.6bc	0.67c	50.6b	18.7ab	161.5ab
	T_5_: SRI-SWI	41.0bc	15.6b	91.8b	171.2ab	13.6ab	145.8c	0.71ab	51.3b	16.3bc	169.4ab
	T_6_: CTDSR-ZTW	46.8b	16.8ab	95.3b	168.8ab	13.4ab	146.7c	0.75a	50.3b	18.3ab	145.8bc
	T_7_: ZTDSR-ZTW	55.7a	18.4a	106.3a	178.1a	14.8a	173.6ab	0.77a	59.2a	20.3a	126.0d
10-20 cm	T_1_: RPTR-CTW	35.3c	9.2c	50.8b	137.9b	9.4b	127.6c	0.60b	49.8c	13.6b	271.7ab
	T_2_: LPTR-CTLW	41.6b	9.1c	52.6b	140.9a	11.0a	128.4c	0.63b	51.9c	13.3b	286.6a
	T_3_: MTNPR-ZTW	43.9b	11.6a	64.3a	159.5a	9.7b	144.3abc	0.67ab	68.6a	17.4a	260.0bc
	T_4_: MTZTR-ZTW	41.5b	10.2b	61.5a	157.5a	9.4b	142.2bc	0.65ab	59.85b	16.7a	256.6bc
	T_5_: SRI-SWI	39.6b	9.9bc	53.8b	143.2a	9.6b	139.9a	0.64ab	57.9b	17.1a	261.7bc
	T_6_: CTDSR-ZTW	43.7b	10.8ab	61.4a	141.1a	11.3a	133.4bc	0.68ab	62.4b	17.3a	243.3cd
	T_7_: ZTDSR-ZTW	48.0a	11.7a	67.3a	150.0a	11.4a	145.5ab	0.69a	69.9a	19.1a	226.6d

*FDA = fluorescein diacetate; DHA = dehydrogenase; MBC = microbial biomass carbon; Av. N = available nitrogen; Av. P = available phosphorus; Av. K = available potassium; O.C = organic carbon; MAS = macro aggregate stability; AWC = available water capacity; SPR = soil penetration resistance

*Means in a column followed by the same letter are not significantly different at *p* < 0.05 according to the Duncan’s multiple range test (n = 3)

* RPTR-CTW: Random puddled transplanted rice (RPTR) - Conventional till broadcasted wheat (CTW) - ZT Greengram

LPTR-CTLW: Puddled line transplanted rice (LPTR)- Conventional tillage line sown wheat (CTLW) - ZT Greengram

MTNPR-ZTW: Machine transplanted non-puddled rice (MTNPR) - Zero-till wheat (ZTW) - ZT Greengram

SRI-SWI: System of rice intensification (SRI) - System of wheat intensification (SWI) - ZT Greengram

CTDSR-ZTW: Conventional till direct seeded rice (CTDSR) - ZTW - ZT Greengram

ZTDSR-ZTW: Zero-till direct seeded rice (ZTDSR) - ZTW - ZT Greengram

The AWC was significantly (*p* < 0.05) highest in the soil with treatment T_7_ and remained at par with T_3_, T_4_ and T_6_ (Table 2). The AWC of soil got improvised under T_7_ (36.2%) and T_3_ (27.5%) in 0-10 cm compared to T_2_ due to surface retention of crop residue input and zero/reduced tillage practices after completion of 4^th^ year (Table 2). A similar pattern existed in the deeper soil layer (10-20 cm). The result of contrast analysis revealed significant difference i.e. higher moisture retention in treatments where residues were left on the surface instead of incorporation into the soil. Similarly, AWC values were statistically (*p* < 0.05) different between CT/ RT, CT/ ZT and RT/ ZT at 0-10 cm but no substantial difference between RT/ZT was observed at 10-20 cm (Table 3).

SPR was significantly different in treatments due to tillage and residue management in both soil depths (Table 2). The SPR was augmented with an increase in soil depth for all of the treatments. Different treatments had significant (*p* < 0.05) effect on soil penetration resistance (SPR) in both soil layers. The SPR under T_7_ was lower by 1.41 and 1.27-folds in 0-10 and 10-20 cm soil profile, respectively, than conventional practices at the end of 4^th^ year (Table 2).

### 3.2 Soil chemical indicators

Crop establishment involving different tillage and residue management practices in rice and wheat significantly affected the soil chemical properties in different soil layers (0-10 and 10-20 cm) (Table 2). The soil organic carbon, an indicator of soil fertility, was positively and significantly (*p* < 0.05) affected by the tillage and residue management practices (Table 3). Retention of crop residues on soil surface had substantially higher OC than that of residue incorporation. In our study, we observed that zero tillage in combination with crop residue (CR) retention increased the OC (1.01 to 1.18-fold) as compared to conventional till plots (RPTR-CTW & LPTR-CTW), with rice crop residue incorporated into the soil. Residue retention with zero-till in treatment T_7_ showed an increase of 18.46% in OC in the upper layer of soil (0-10 cm) and an increase of 15% in 10-20 cm compared to conventional tillage practice T_1_.

Availability of nutrients was significantly (*p* < 0.05) affected in different treatments (Table 2). The availability of N in 0-10 cm depth with complete zero tillage practice T_7_ was found to be the highest but at par with T_3_, T_5_ and T_6_ as compared to T_1_, T_2_ and T_4_. With the increase in soil depth, the content of available nutrients (N, P and K) was low for all treatments. Results also indicate that an accumulation of P and K with zero/reduced tillage with all three crop residues (rice, wheat and greengram) either retained or incorporated (T_7_, T_6_ and T_3_) occurs in the upper soil layers and depletion in the deepest sampled soil layer. T_7_ resulted in a 1.32-fold increase of P in 0-10 cm depth whereas a 1.26-fold increase in K in T_3_ was observed compared to T_1._ However, in 10-20 cm depth the increase was 1.21-fold for P and 1.14-fold for K in T_7_ as compared to T_1_.

### 3.3 Soil biological indicators

The range of MBC value as overall, observed between 50.8 to 106.3 μg C g^-1^ soils (Table 2). The results indicated a significant (*p* < 0.05) difference in the level of soil MBC between various treatments of crop residue retention and tillage practices. The MBC readings were significantly greater in the treatments in which CRs retained with zero/reduced tillage practices i.e. in T_7_ and T_3_. With respect to MBC in 0-10 cm depth the treatment could be arranged in the order of T_7_ (106.3 μg g^-1^) > T_3_ (98.1 μg g^-1^) > T_6_ (95.3 μg g^-1^) > T_4_ (94 μg g^-1^) > T_5_ (91.8 μg g^-1^) > T_2_ (71.8 μg g^-1^) > T_1_ (67.8 μg g^-1^). Inputs of readily metabolizable and hydrolyzable C and N in crop residues are the most important factors contributing to the MBC increase in T_7_ and T_3_ in combination with less soil disturbance due to zero tillage. Similarly, higher values of dehydrogenase and FDA were observed in T_7_. FDA values ranged between 37.4 in T_1_ to 55.7 mg kg^-1^ soil hr^-1^ in T_7_ treatment at two soil depth. Likewise, DHA activity ranged from 12 in T_1_ to 18.4 μg g ^−1^ soil h^−1^ in T_7_. Continuous addition of crop residue on the surface and ZT caused an increase of 1.57-fold, 1.41-fold and 1.53-fold in MBC, FDA and DHA activities respectively, over T_1_ treatments. All these biological soil properties were significantly (*p* < 0.05) different under Ri and Rr residue treatments and CT and ZT tillage practice treatment mean groups. However, there was no significant (p < 0.05) difference observed between RT and ZT for these parameters (Table 3).

### 3.4 Soil quality index and crop yield

**Fig. 1:**
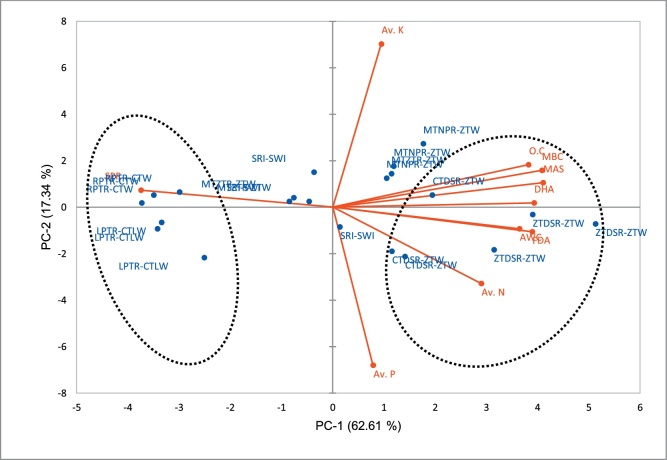
Results obtained by Principal Component Analysis. PC-1 x PC-2 biplots: scores (treatments) are represented by blue colours and loadings (soil properties) by red. *FDA = fluorescein diacetate; DHA = dehydrogenase; MBC = microbial biomass carbon; Av. N = available nitrogen; Av. P = available phosphorus; Av. K = available potassium; O.C = organic carbon; MAS = macro aggregate stability; AWC = available water capacity; SPR = soil penetration resistance *RPTR-CTW: Random puddled transplanted rice (RPTR) - Conventional till broadcasted wheat (CTW) - ZT Greengram LPTR-CTLW: Puddled line transplanted rice (LPTR)- Conventional tillage line sown wheat (CTLW) - ZT Greengram MTNPR-ZTW: Machine transplanted non-puddled rice (MTNPR) - Zero-till wheat (ZTW) - ZT Greengram SRI-SWI: System of rice intensification (SRI) - System of wheat intensification (SWI) - ZT Greengram CTDSR-ZTW: Conventional till direct seeded rice (CTDSR) - ZTW - ZT Greengram ZTDSR-ZTW: Zero-till direct seeded rice (ZTDSR) - ZTW - ZT Greengram

Physical, chemical and biological properties of mean soil layer (0-20 cm) were subjected to PCA. The result showed that 79.96% of the total variance was explained by two principal components (PCs) (Table 4) with eigenvalues greater than one. The PC-1 and PC-2 described 62.61 and 17.3% of the variability, respectively. Since the majority of the data information explained by PC-1 allows differentiation between different management practices applied to plots. In biplot, the location of soil properties and different treatments scattered in different regions of PCA axes is on the basis of the correlation coefficient between variables, treatments occupying opposite places in the diagram i.e. in the opposite direction of an axis show distinct variations (Fig. 1). The variables that are largely accountable for this variation can be observed by simultaneously projecting scores (treatments) and loading (variables) onto PC-1 axis (Fig. 1). Variables like FDA, DHA, MBC, SOC, AWC and MAS were positively correlated (close) to plots where zero/reduced tillage practices were adopted, whereas all these variables (soil properties) were negatively correlated to conventional tillage treatments (Fig. 1). Another important observation recorded that negative correlation was observed between SPR and zero/reduced tillage practice.

**Table 4:** Results of principal component analysis showing principal components (PC) with their Eigenvalues and proportion of variance (in percent) explained, along with rotated factor loadings and communalities of soil attributes.

Soil properties	PC-1	PC-2	Communalities
MAS	0.956	−0.114	0.836
MBC	0.951	−0.179	0.834
DHA	0.913	−0.008	0.936
FDA	0.903	0.143	0.615
OC	0.891	−0.21	0.726
SPR	−0.869	−0.104	0.787
AWC	0.846	0.126	0.839
Av.N	0.667	0.412	0.926
Av.K	0.233	−0.856	0.731
Av.P	0.17	0.835	0.766
Eigenvalues	6.26	1.73	
Variance explained (%)	62.61	17.34	
Cumulative Variance explained (%)	62.61	79.96	

*FDA = fluorescein diacetate; DHA = dehydrogenase; MBC = microbial biomass carbon; Av. N = available nitrogen; Av. P = available phosphorus; Av. K = available potassium; O.C = organic carbon; MAS = macro aggregate stability; AWC = available water capacity; SPR = soil penetration resistance

**Fig. 2:**
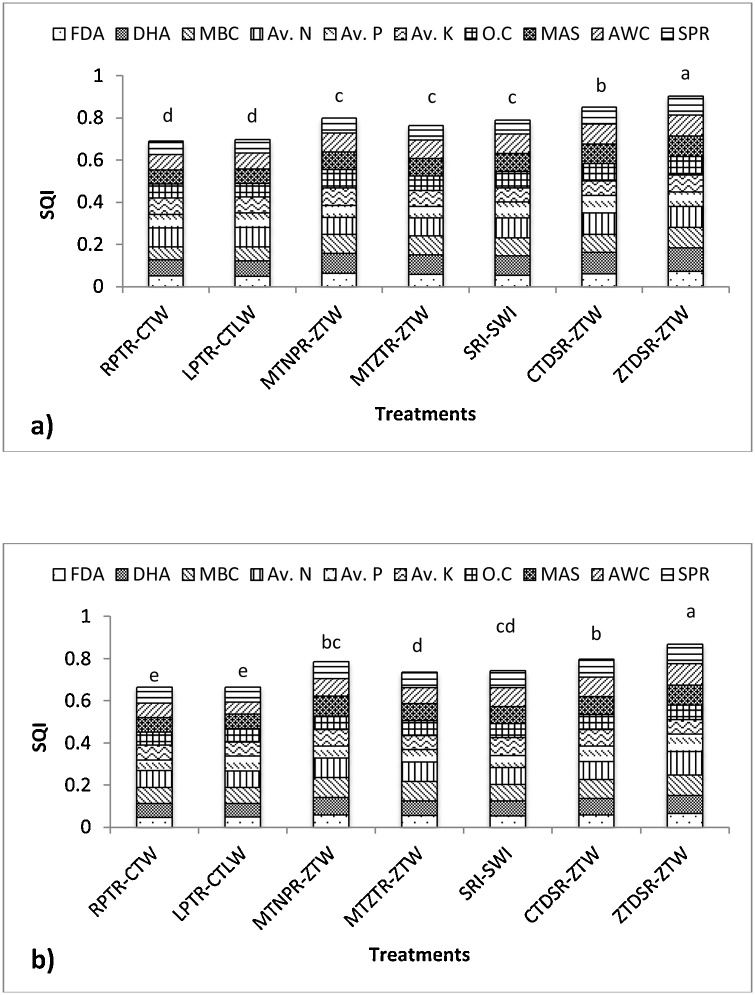
Contributions of individual soil indicator parameter to overall soil quality index under different treatments (a) 0–10 cm depth; (b) 10–20 cm depth. Diﬀ ;erent letters above the bars indicate signiﬁcant diﬀ ;erences at *p* < 0.05 within a soil layer. *FDA = fluorescein diacetate; DHA = dehydrogenase; MBC = microbial biomass carbon; Av. N = available nitrogen; Av. P = available phosphorus; Av. K = available potassium; O.C = organic carbon; MAS = macro aggregate stability; AWC = available water capacity; SPR = soil penetration resistance

In order to differentiate the mean values for different treatments, SQI scores were subjected to ANOVA. As indicated in Fig. 2, the percentage contribution of the selected indicators to form the treatment-wise SQI has been presented. The SQI was significantly higher for the 0-10 cm (0.69 – 0.90) than the 10-20 cm (0.66 – 0.86). For 0-10 cm soil layer, the SQI followed the order: T_7_ (0.90) > T_6_ (0.85) > T_3_ (0.80) > T_5_ (0.79) > T_4_ (0.76) > T_2_ (0.70) > T_1_ (0.69) and for 0-20 cm soil layer: T_7_ (0.86) > T_6_ (0.80) > T_3_ (0.79) > T_4_ (0.74) ≥ T_5_ (0.74) > T_2_ (0.67) ≥ T_1_ (0.66).

**Fig. 3:**
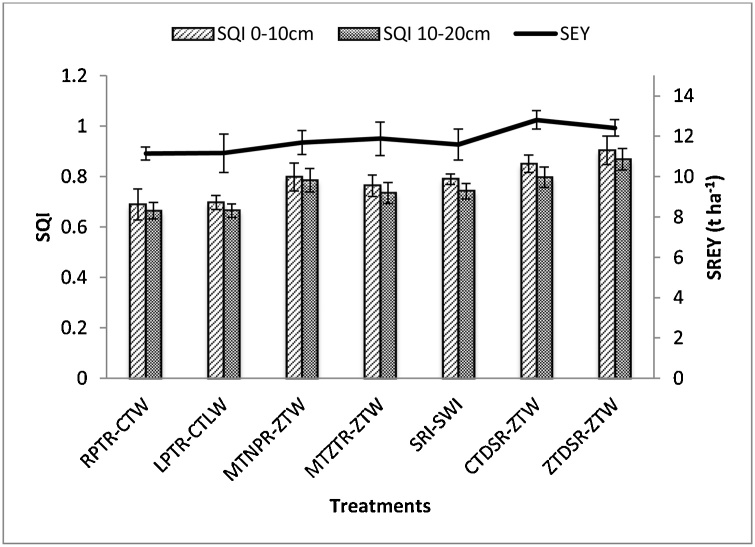
Soil quality index (SQI) and system rice equivalent yield (SREY) at 0-10 cm and 10-20 cm under different treatments.

**Fig. 4:**
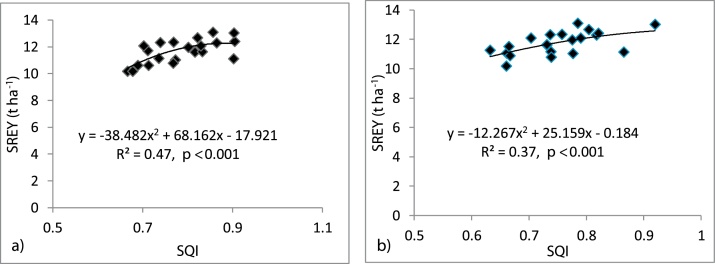
Relationships between soil quality index (SQI) and system rice equivalent yield (SREY) at 0-10 cm (a) and 10-20 cm (b).

Statistically significant higher SREY were observed in T_6_ treatment compared to others with the decreasing order as follows: T_6_ > T_7_ > T_3_ > T_4_ > T_5_ > T_2_ > T_1_ (Fig. 3). The calculated SQIs were validated by correlation with the SREY. After plotting graph between SQI values and SREY for both the soil layers, we got a positive and significant correlation between SREY and SQI. The calculated SQI relates well to SREY (*R^2^* = 0.47, *p* < 0.001) at 0-10 cm and (*R^2^* = 0.37, *p* < 0.001) at 10-20 cm soil depth (Fig.4). Higher scores of SQI signify better soil quality and vice versa. The minimum SREY was 11.14 t ha^-1^ in T_1_ (though it was statistically at par with other treatments except for T_6_) at SQI value of 0.68 (0-10 cm) and 0.66 (10-20 cm) whereas, the highest yield of 12.81 t ha^-1^ was recorded in T_6_ at SQI value of 0.86 (0-10 cm) and 0.81 (10-20 cm) (Fig. 4). Maximum SQI value 0.94 (0-10 cm) and 0.86 (10-20 cm) was observed in T_7_ treatment with 12.41 t ha^-1^ SREY which was significantly at par with T_6_.

## 4 Discussion

### 4.1 Soil physical indicators

Soil physical parameter like, aggregate stability is considered as the main soil physical indicator as it depicts the soil’s ability to resist mechanical disruption that may lead to soil erosion. It influences other soil properties like soil porosity, soil structure, water holding capacity, organic matter stability and elemental bio-cycling (Ghildyal and Tripathi, 1987; Gelaw et al., 2015). The amount of carbon in soil strongly influences the aggregate stability. Doran (1987) observed less oxidative soil environment under zero tillage practice than conventional tillage, which favours the carbon stabilization in such soils. We observed that treatments having ZT and RT with residue retention on the surface enabled the safety of organic matter from microbial degradation due to lesser soil disruption, which in turn favoured the generation of physically stable macro-aggregates (Mondal et al., 2019). Similar observations have been reported by Sarker et al. (2018). Tillage caused the breakdown of macro-aggregate to finer aggregates and facilitated the degradation of exposed organic matter present in the soil, thus reduced soil aggregation, making the soil more sensitive to wind or water erosion in conventional tillage treatments such as T_1_, T_2_ and T_5_.

AWC is the water held in soil between its field capacity and permanent wilting point. Water availability is an important indicator since plant growth and soil biological activities depend on water for hydration and delivery of nutrients in solution (Basche et al., 2016). Conservation of soil moisture due to less tillage and residue retention is one of the significant advantages in conservation agriculture. Crop residues retained on the soil surface under zero tillage shade the soil and serve as a vapour barrier against moisture loss from the soil. Mahboubi et al. (1993) reported a higher AWC under crop residue and no-till treatment. Similar observations were made by Mulumba and Lal (2008).

SPR also plays an important role in plant growth by affecting root penetration and growth in soil (Lampurlanés and Cantero-Martinez, 2003). Penetration resistance in a given soil is directly related to bulk density and inversely related to soil water content (Sharma and De Datta, 1986; Unger and Jones, 1998). In our study, the lowest SPR values were generally recorded at T_7_ due likely to the higher organic carbon and water content of the soil at the time of sampling. Intensive tillage operation increases compaction and soil bulk density and thereby increases the cone penetrometer resistance. Franzluebbers (2002) reported lower penetration resistance of intact soil under long-term ZT because of a high percentage of SOC as compared to the long-term CT with a low SOC. The other reason for lower penetration resistance under ZT treatments might be because of a lower amount of traffic due to absensence of tillage under ZT as compared to CT (McFarland et al., 1990). Increased penetration resistance with increasing depth either in ZT/RT or CT was observed by other researchers (McFarland et al., 1990; Gathala et al., 2017). In contrary to our results, other researchers reported increased penetration resistance in reduced and especially ZT treatments compared to conventional tillage methods (Schwartz et al. 2003; Dalal 1992; Moret and Arrúe, 2007; Alvarez et al. 2009).

### 4.2 Soil chemical indicators

SOC content was found to be higher in ZT/RT treatments (T_7_, T_6_ and T_3_) than conventional practice (T_1_ and T_2_) at both soil depths. Though in treatment T_4_, zero tillage practice was involved, comparatively less OC content was found due to the removal of greengram residue as it was difficult to run a machine during ZT rice transplanting. In this study, T_7_ emerged as the best option in improving the soil organic carbon status for our experimental crops. Thus the present study suggests that retention of one-third rice residue on the surface in combination with zero tillage DSR is helpful in sequestering OC. Choudhury et al. (2014) confirmed that DSR and zero-tilled wheat combined with CR retention is an appropriate management practice to improve sequestration since it could increase the total content of SOC by 33.6% as compared to conventional tillage. Zero tillage in combination with or without crop residues increases SOC, limits soil disturbance, improves soil aggregation and reduces the disruptive effect of tillage on SOC loss through increased soil microbial respiration (Six et al., 2000; Balesdent et al., 2000).

Residue placement is among the major factors affecting the nutrient availability in conservation agriculture. In the case of ZT and RT with surface retained residue, a higher amount of available N was observed due to higher N-immobilization. This increased the long-term conservation of soil and fertilizer N due to decreased erosion losses and the build-up of mineralizable organic N on the soil surface. Further, a higher amount of P and K was found in surface soil in treatments where crop residues were retained and could be ascribed to leaching of soluble forms of these elements (P and K) (Schoenau and Campbell, 1996; Willson et al., 2001). Neugschwandtner et al. (2014) reported higher concentration of available P and K in reduced tillage treatment in the top soil layers as compared to conventional tillage. Wei et al. (2015) studied the effect of the incorporation of wheat straw on the availability of nutrients and found an increase (9.1-30.5%) in the available N contents at 0–40 cm soil layers with straw incorporation treatments while available P and K were increased by 9.8–69.5% and 10.3–27.3%, respectively. The addition of crop residues to the soil and its consequent decay stock up the content of organic matter in the soil and also supplies essential nutrients after mineralization (N, P, S and Si) (Surekha et al., 2003), which augment the microbial and enzymatic activity in the soil with subsequent nutrient transformation. Thus the combined treatment of ZT with crop residue retention and inclusion of legume in cropping system could be an effective strategy to improve the availability of soil nutrients (Roldan et al., 2003).

### 4.3 Soil biological indicators

Soil microbiological activities (MBC, FDA and DHA) were found significantly higher where ZT was adopted along with surface retention of CR (Garcia et al., 1997; Roldan et al., 2003). Signiﬁcantly higher MBC, FDA and DHA activities in the surface than the sub-surface soil was observed due to less availability of crop residue in lower soil depth. Similar results are reported by others (Sharma et al., 2005; Mukhopadhyay et al., 2016; Samal et al., 2017). The retention of rice residue in treatments had a positive effect on soil as this increased soil organic carbon and water availability which ultimately improved the soil microbial parameters.

### 4.4 Soil quality and crop yield

Lower values of SQI in T_1_ and T_2_ are mainly due to conventional tillage practice and in T_4_ due to removal of greengram residue mainly degraded the biological (MBC, FDA and DHA), physical soil health and reduced soil OC content in such treatments which in turns reduced the system yield. The higher yield recorded in T_7_ and T_6_ highlights the beneficial role of ZT/RT and surface residue retention practices. These results showed that under identical nutrient management conditions, tillage based crop establishment systems with different residue management determine the soil physical and biochemical indices. Overall the retention of organic matter and reduced/zero tillage were found to influence the soil properties and overall system yield.

A similar result was observed by Das et al. (2016) where the treatment receiving crop residue along with NPK showed maximum SQI value due to significant improvement in soil physical properties. Mohanty et al. (2007) showed that the most positive effect on soil quality was due to the use of ZT in wheat. They also reported that the long-term sustainability and higher productivity could be achieved in CT with direct seeding of rice in combination with residues retention. Specific studies have also shown higher wheat yields under ZT (with and without residue retention) compared to traditional tillage in the eastern IGP region (Singh et al., 2020; Nandan et al., 2018). Likewise, Samal et al. (2017) observed significantly higher system productivity in terms of rice equivalent yield under ZT compared to the conventional tillage.

## 5 Conclusions

The return of crop residues has a positive impact on soil quality as reflected in such indices as soil organic carbon content, MBC, FDA, DHA, AWC, SPR and water-stable aggregates. Clearly, a positive effect of zero-tillage or reduced tillage on crop yields and associated residue inputs contributed to overall soil quality and fertility. The relationship between selected soil properties and system yield showed a high correlation indicated that the improvements observed in soil properties as a result of the application of residues with reduced tillage contributed to the higher system yield. We, therefore, conclude that the improved soil structure, microbial activity and soil enzyme activities in intensively cultivated rice-wheat crops could be achieved by implementing zero till technology and by applying crop residues, including legumes, in crop sequences. In particular, the joint management of ZT in rice and wheat, the retention of one -third crop residues on the soil surface and the planting of legumes (ZTDSR + ZTW) can be regarded as an effective technology for the implementation of sustainable agriculture in the EIGP due to its rapid improvement in soil quality.

## Declaration of Competing Interest

The authors report no declarations of interest.

## References

[bib0005] Al-Maliki S., Scullion J. (2013). Interactions between earthworms and residues of differing quality affecting aggregate stability and microbial dynamics. Appl. Soil Ecol..

[bib0010] Alvarez C.R., Taboada M.A., Gutierrez Boem F.H., Bono A., Fernandez P.L., Prystupa P. (2009). Topsoil properties as affected by tillage systems in the Rolling Pampa region of Argentina. Soil Sci. Soc. Am. J..

[bib0015] Andrews S., Karlen D., Mitchell J. (2002). A comparison of soil quality indexing methods for vegetable production systems in Northern California. Agric. Ecosyst. Environ..

[bib0020] Balesdent J., Chenu C., Balabane M. (2000). Relationship of soil organic matter dynamics to physical protection and tillage. Soil Tillage Res..

[bib0025] Basche A.D., Kaspar T.C., Archontoulis S.V., Jaynes D.B., Sauer T.J., Parkin T.B., Miguez F.E. (2016). Soil water improvements with the long-term use of a winter rye cover crop. Agr. Water Manage..

[bib0030] Bohra J.S., Kumar R. (2015). Effect of crop establishment methods on productivity, profitability and energetics of rice (Oryza sativa)-wheat (Triticum aestivum) system. Indian J. Agr. Sci..

[bib0035] Brejda J.J., Moorman T.B., Karlen D.L., Dao T.H. (2000). Identification of regional soil quality factors and indicators: I. Central and southern high plains. Soil Sci. Soc. Am. J..

[bib0040] Choudhury S.G., Srivastava S., Singh R., Chaudhari S.K., Sharma D.K., Singh S.K., Sarkar D. (2014). Tillage and residue management effects on soil aggregation, organic carbon dynamics and yield attribute in rice–wheat cropping system under reclaimed sodic soil. Soil Tillage Res..

[bib0045] Dalal R.C. (1992). Long term trends in total nitrogen of a vertisol subjected to zero-tillage, nitrogen application and stubble retention. Soil Res..

[bib0050] Das B., Chakraborty D., Singh V.K., Ahmed M., Singh A.K., Barman A. (2016). Evaluating fertilization effects on soil physical properties using a soil quality index in an intensive rice-wheat cropping system. Pedosphere..

[bib0055] Devi S., Gupta C., Jat S.L., Parmar M.S. (2017). Crop residue recycling for economic and environmental sustainability: The case of India. Open Agriculture.

[bib0060] Doran J., Coleman D., Bezdicek D., Stewart B. (1994). A framework for evaluating physical and chemical indicators of soil quality.

[bib0065] Doran J.W. (1987). Microbial biomass and mineralizable nitrogen distributions in no-tillage and plowed soils. Biol. Fertil. Soils..

[bib0070] Doran J.W. (2002). Soil health and global sustainability: translating science into practice. Agric. Ecosyst. Environ..

[bib0075] Franzluebbers A.J. (2002). Water infiltration and soil structure related to organic matter and its stratification with depth. Soil Tillage Res..

[bib0080] Garcia C., Hernandez T., Roldan A. (1997). Changes in microbial activity after abandonment of cultivation in a semiarid Mediterranean environment. J. Environ. Qual..

[bib0085] Gathala M.K., Jat M.L., Saharawat Y.S., Sharma S.K., Singh Y., Ladha J.K. (2017). Physical and chemical properties of a sandy loam soil under irrigated rice-wheat sequence in the Indo-Gangetic Plains of South Asia. J Ecosys Ecograph..

[bib0090] Gelaw A.M., Singh B.R., Lal R. (2015). Soil quality indices for evaluating smallholder agricultural land uses in northern Ethiopia. Sustainability..

[bib0095] Ghildyal B.P., Tripathi R.P. (1987). Soil Physics.

[bib0100] Ghosh B.N., Dogra P., Sharma N.K., Bhattacharyya R., Mishra P.K. (2015). Conservation agriculture impact for soil conservation in maize–wheat cropping system in the Indian sub-Himalayas. Inter. Soil Water Conserv. Res..

[bib0105] Green V.S., Stott D.E., Diack M. (2006). Assay for fluorescein diacetate hydrolytic activity: optimization for soil samples. Soil Biol. Biochem..

[bib0110] Hanway J.J., Heidel H. (1952). Soil analysis methods as used in Iowa state college soil testing laboratory. Iowa agriculture.

[bib0115] Islam K.R., Weil R.R. (2000). Land use effects on soil quality in a tropical forest ecosystem of Bangladesh. Agric. Ecosyst. Environ..

[bib0120] Karlen D.L., Mausbach M.J., Doran J.W., Cline R.G., Harris R.F., Schuman G.E. (1997). Soil quality: a concept, definition, and framework for evaluation (a guest editorial). Soil Sci. Soc. Am. J..

[bib0125] Lal R. (2015). Restoring soil quality to mitigate soil degradation. Sustainability..

[bib0130] Lampurlanés J., Cantero-Martinez C. (2003). Soil bulk density and penetration resistance under different tillage and crop management systems and their relationship with barley root growth. Agr. J..

[bib0135] Larson W.E., Pierce F.J. (1991). Conservation and enhancement of soil quality. Evaluation of Sustainable Land Management in the Developing World.

[bib0140] Li Y., Chang S.X., Tian L., Zhang Q. (2018). Conservation agriculture practices increase soil microbial biomass carbon and nitrogen in agricultural soils: A global meta-analysis. Soil Biol. Biochem.

[bib0145] Liu Y.L., Chang K.T., Stoorvogel J., Verburg P., Sun C.H. (2012). Evaluation of agricultural ecosystem services in fallowing land based on farmers’ participation and model simulation. Paddy and Water Environment.

[bib0150] Liu Z., Rong Q., Zhou W., Liang G. (2017). Effects of inorganic and organic amendment on soil chemical properties, enzyme activities, microbial community and soil quality in yellow clayey soil. PloS one.

[bib0155] Mahajan A., Gupta R.D., Mahajan A., Gupta R.D. (2009). The Rice–Wheat Cropping System. Integrated nutrient management (INM) in a sustainable rice-wheat cropping system..

[bib0160] Mahboubi A.A., Lal R., Faussey N.R. (1993). Twenty-eight years of tillage effects on two soils in Ohio. Soil Sci. Soc. Am. J..

[bib0165] Masto R.E., Chhonkar P.K., Singh D., Patra A.K. (2007). Soil quality response to long-term nutrient and crop management on a semi-arid Inceptisol. Agric. Ecosyst. Environ..

[bib0170] Mbuthia L.W., Acosta-Martínez V., DeBruyn J., Schaeffer S., Tyler D., Odoi E., Mpheshea M., Walker F., Eash N. (2015). Long term tillage, cover crop, and fertilization effects on microbial community structure, activity: Implications for soil quality. Soil Biol. Biochem..

[bib0175] McFarland M.L., Hons F.M., Lemon R.G. (1990). Effects of tillage and cropping sequence on soil physical properties. Soil Tillage Res..

[bib0180] Mohanty M., Painuli D.K., Misra A.K., Ghosh P.K. (2007). Soil quality effects of tillage and residue under rice–wheat cropping on a Vertisol in India. Soil Tillage Res.

[bib0185] Mondal M., Kumar S., Haris A.A., Dwivedi S.K., Bhatt B.P., Mishra J.S. (2016). Eﬀ ;ect of diﬀ ;erent rice establishment methods on soil physical properties in drought-prone, rainfed lowlands of Bihar. India. Soil Res..

[bib0190] Mondal S., Poonia S.P., Mishra J.S., Bhatt B.P., Karnena K.R., Saurabh K., Kumar R., Chakraborty D. (2019). Short‐term (5 years) impact of conservation agriculture on soil physical properties and organic carbon in a rice–wheat rotation in the Indo‐Gangetic plains of Bihar. Eur J Soil Sci..

[bib0195] Montgomery D.R. (2007). Soil erosion and agricultural sustainability. Proc. Natl. Acad. Sci..

[bib0200] Moret D., Arrúe J.L. (2007). Dynamics of soil hydraulic properties during fallow as affected by tillage. Soil Tillage Res..

[bib0205] Mukhopadhyay S., Masto R.E., Yadav A., George J., Ram L.C., Shukla S.P. (2016). Soil quality index for evaluation of reclaimed coal mine spoil. Sci. Total Environ..

[bib0210] Mulumba L.N., Lal R. (2008). Mulching effects on selected soil physical properties. Soil Tillage Res..

[bib0215] Nandan R., Singh S.S., Kumar V., Singh V., Hazra K.K., Nath C.P., Malik R.K., Poonia S.P., Solanki C.H. (2018). Crop establishment with conservation tillage and crop residue retention in rice-based cropping systems of Eastern India: yield advantage and economic benefit. Paddy Water Environ..

[bib0220] Neugschwandtner R.W., Liebhard P., Kaul H.P., Wagentristl H. (2014). Soil chemical properties as affected by tillage and crop rotation in a long-term field experiment. Plant Soil Environ..

[bib0225] Nunan N., Morgan M.A., Herlihy M. (1998). Ultraviolet absorbance (280 nm) of compounds released from soil during chloroform fumigation as an estimate of the microbial biomass. Soil Biol. Biochem..

[bib0230] Panigrahy S., Upadhyay G., Ray S.S., Parihar J.S. (2010). Mapping of cropping system for the Indo-Gangetic plain using multi-date SPOT NDVI-VGT data. J. Indian Soc. Remote..

[bib0235] Parihara C.M., Yadav M.R., Jata S.L., Singh A.K., Kumara B., Pradhan S., Chakraborty D., Jat M.L., Jat R.K., Saharawat Y.S., Yadav O.P. (2016). Long term eﬀ ;ect of conservation agriculture in maize rotations on total organic carbon, physical and biological properties of a sandy loam soil in north-western Indo-Gangetic Plains. Soil Tillage Res..

[bib0240] Piper C.S. (1966). ‘Soil and plant analysis.’.

[bib0245] Roldan A., Caravaca F., Hernández M.T., Garcıa C., Sánchez-Brito C., Velásquez M., Tiscareno M. (2003). No-tillage, crop residue additions, and legume cover cropping effects on soil quality characteristics under maize in Patzcuaro watershed (Mexico). Soil Tillage Res..

[bib0250] Samal S.K., Rao K.K., Poonia S.P., Kumar R., Mishra J.S., Prakash V., Mondal S., Dwivedi S.K., Bhatt B.P., Naik S.K., Choubey A.K. (2017). Evaluation of long-term conservation agriculture and crop intensification in rice-wheat rotation of Indo-Gangetic Plains of South Asia: Carbon dynamics and productivity. Eur. J. Agron..

[bib0255] Sanchez-Maranon M., Soriano M., Delgado G., Delgado R. (2002). Soil quality in Mediterranean mountain environments: Effects of land use change. Soil Sci. Soc. Am. J..

[bib0260] Sarker J.R., Singh B.P., Cowie A.L., Fang Y., Collins D., Badgery W., Dalal R.C. (2018). Agricultural management practices impacted carbon and nutrient concentrations in soil aggregates, with minimal influence on aggregate stability and total carbon and nutrient stocks in contrasting soils. Soil Tillage Res..

[bib0265] Schoenau J.J., Campbell C.A. (1996). Impact of crop residues on nutrient availability in conservation tillage systems. Can. J. Plant Sci..

[bib0270] Schwartz R.C., Evett S.R., Unger P.W. (2003). Soil hydraulic properties of cropland compared with reestablished and native grassland. Geoderma..

[bib0275] Sharma K.L., Mandal U.K., Srinivas K., Vittal K.P.R., Mandal B., Grace J.K., Ramesh V. (2005). Long-term soil management effects on crop yields and soil quality in a dryland Alfisol. Soil Tillage Res..

[bib0280] Sharma P.K., De Datta S.K. (1986). Physical properties and processes of puddled rice soils. Adv. Soil Sci..

[bib0285] Shekhawat K., Rathore S.S., Kandpal B.K., Premi O.P., Singh D., Chauhan B.S. (2016). Crop establishment techniques affect productivity, sustainability, and soil health under mustard-based cropping systems of Indian semi-arid regions. Soil Tillage Res..

[bib0290] Singh M., Kumar P., Kumar V., Solanki I.S., McDonald A.J., Kumar A., Poonia S.P., Kumar V., Ajay A., Kumar A., Singh D.K. (2020). Intercomparison of crop establishment methods for improving yield and profitability in the rice-wheat system of Eastern India. Field Crops Res..

[bib0295] Singh V.K., Dwivedi B.S., Singh S.K., Mishra R.P., Shukla A.K., Rathore S.S., Shekhawat K., Majumdar K., Jat M.L. (2018). Effect of tillage and crop establishment, residue management and K fertilization on yield, K use efficiency and apparent K balance under rice-maize system in north-western India. Field Crops Res..

[bib0300] Six J., Elliott E.T., Paustian K. (2000). Soil macroaggregate turnover and microaggregate formation: a mechanism for C sequestration under no-tillage agricultural. Soil Biol. Biochem..

[bib0305] Subbiah B.V., Asija G.L. (1956). A rapid procedure for the determination of available nitrogen in soils. Current Science.

[bib0310] Surekha K., Kumari A.P., Reddy M.N., Satyanarayana K., Cruz P.S. (2003). Crop residue management to sustain soil fertility and irrigated rice yields. Nutr. Cycl. Agroecosys..

[bib0315] Tabatabai M.A., Bremner J.M. (1969). Use of *p*-nitrophenyl phosphate for assay of soil phosphatase activity. Soil Biol. Biochem..

[bib0320] Tadesse T., Dechassa N., Bayu W., Gebeyehu S. (2013). Effects of farmyard manure and inorganic fertilizer application on soil physico-chemical properties and nutrient balance in rain-fed lowland rice ecosystem. Am. J. Plant Sci..

[bib0325] Tisdall J., Oades J. (1982). Organic-matter and water-stable aggregates in soils. J. Soil Sci.

[bib0330] Unger P.W., Jones O.R. (1998). Long-term tillage and cropping systems affect bulk density and penetration resistance of soil cropped to dryland wheat and grain sorghum. Soil Tillage Res..

[bib0335] Walkley A., Black I.A. (1934). An examination of the Degtjareff method for determining soil organic matter and a proposed modification of the chromic acid titration method. Soil Sci..

[bib0340] Watanabe F.S., Olsen S.R. (1965). Test of ascorbic acid method for determining phosphorus in water and sodium bicarbonate extracts of soil. Soil Sci. Soc. Am. Pro..

[bib0345] Wei T., Zhang P., Wang K., Ding R., Yang B., Nie J., Jia Z., Han Q. (2015). Effects of wheat straw incorporation on the availability of soil nutrients and enzyme activities in semiarid areas. PLoS One..

[bib0350] Willson T.C., Paul E.A., Harwood R.R. (2001). Biologically active soil organic matter fractions in sustainable cropping systems. Appl. Soil Ecol..

[bib0355] Zhang S., Chen X., Jia S., Liang A., Zhang X., Yang X., Wei S., Sun B., Huang D., Zhou G. (2015). The potential mechanism of long-term conservation tillage effects on maize yield in the black soil of Northeast China. Soil Tillage Res..

